# Effects of Benzo[a]pyrene on Human Sperm Functions: An In Vitro Study

**DOI:** 10.3390/ijms241914411

**Published:** 2023-09-22

**Authors:** Giulia Traini, Lara Tamburrino, Maria Emanuela Ragosta, Giulia Guarnieri, Annamaria Morelli, Linda Vignozzi, Elisabetta Baldi, Sara Marchiani

**Affiliations:** 1 Department of Experimental and Clinical Biomedical Sciences “Mario Serio”, University of Florence, 50139 Florence, Italy; giulia.traini@unifi.it (G.T.); linda.vignozzi@unifi.it (L.V.); 2 Andrology, Women’s Endocrinology and Gender Incongruence Unit, Center for Prevention, Diagnosis and Treatment of Infertility, Careggi University Hospital, 50134 Florence, Italy; lara.tamburrino@unifi.it; 3 Department of Experimental and Clinical Medicine, University of Florence, 50139 Florence, Italy; mariaemanuela.ragosta@unifi.it (M.E.R.); giulia.guarnieri@unifi.it (G.G.); a.morelli@unifi.it (A.M.)

**Keywords:** human spermatozoa, Benzo(a)pyrene (BaP), Polycyclic Aromatic Hydrocarbons (PAHs), sperm motility, hyperactivation, spermatozoa able to penetrate in a viscous medium, acrosome reaction, sperm DNA fragmentation, apoptosis, reactive oxygen species

## Abstract

Benzo(a)pyrene (BaP) is considered one of the most dangerous air pollutants for adverse health effects, including reproductive toxicity. It is found both in male and female reproductive fluids likely affecting spermatozoa after the selection process through cervical mucus, a process mimicked in vitro with the swim-up procedure. In vitro effects of BaP (1, 5, 10 µM) were evaluated both in unselected and swim-up selected spermatozoa after 3 and 24 h of incubation. BaP reduced total, progressive and hyperactivated motility and migration in a viscous medium both in swim-up selected and unselected spermatozoa. Viability was not significantly affected in swim-up selected but was reduced in unselected spermatozoa. In swim-up selected spermatozoa, increases in the percentage of spontaneous acrosome reaction and DNA fragmentation were observed after 24 h of incubation, whereas no differences between the control and BaP-treated samples were observed in caspase-3 and -7 activity, indicating no effects on apoptotic pathways. ROS species, evaluated by staining with CellROX^®^ Orange and Dihydroethidium, did not differ in viable spermatozoa after BaP treatment. Conversely, the percentage of unviable ROS-positive spermatozoa increased. Our study suggests that BaP present in male and female genital fluids may heavily affect reproductive functions of human spermatozoa.

## 1. Introduction

Infertility is defined as the inability to conceive after 1 year of regular unprotected sexual intercourse [1] and it is estimated to affect 8–12% of couples in the reproductive age. Male infertility is responsible, alone or in combination, with the female one of approximately 50% of reported cases. Several factors might affect male fertility, including lifestyles, diabetes, obesity, hormonal diseases, testicular trauma, cryptorchidism, varicocele, genitourinary infections, ejaculatory disorders, and chemo- and radio-therapy [2]. Over recent decades, a reduction in semen quality has been reported [3,4], leading to an urgent need to search for the causes and to take actions to prevent such a decline.

Exposure to environmental pollutants has been clearly associated with a range of adverse health effects, including reproductive toxicity [5,6,7]. 

Among the dangerous pollutants for human health are Polycyclic Aromatic Hydrocarbons (PAHs), a large group of organic compounds, composed of two or more fused benzene rings arranged in various configurations. This class of organic chemicals is considered one of the most dangerous air pollutants, mostly because of its carcinogenic and mutagenic characteristics [8,9].

Benzo(a)pyrene (BaP) is the main representative and the most widely investigated PAH. BaP is produced by the incomplete combustion of organic material and is released into the environment from automobile exhausts, cigarette smoke, burning of refuse, industrial emissions, and hazardous waste sites [10]. In mammals, BaP is assimilated by inhalation, through food intake or tobacco smoke, and dermal exposure [11,12,13].

Among the toxic effects of BaP on male reproduction, a decrease in sperm production and motility, an increase in sperm apoptosis, an alteration of Leydig cell steroidogenesis, and an increase in genome-wide mutations have been demonstrated in animal models [14,15,16,17]. The effects of BaP on reproductive fertility status in humans is less investigated; however, some evidence has emerged from in vitro and observational studies, including detrimental effects on central neuroendocrine control of reproduction [18,19]. Men occupationally exposed to high doses of PAHs showed a decreased semen quality with respect to the controls [20,21,22,23]. Recently, Nayak et al. [24] found high levels (about 10,600 ng/mL) of PAHs in semen of unexposed, non-smoking men with normal BMI. In particular, BaP levels were among the 13 investigated PAHs, those that better discriminated between fertile and idiopathic infertile men, reaching levels of 43.37 ± 38.57 ng/mL (about 0.2 µM) in the latter. 

Previous studies demonstrated that in vitro incubation of human spermatozoa with BaP leads to a decreased total and progressive sperm motility, impaired chromatin compaction, DNA damage, increased sperm lipoperoxidation and mitochondrial superoxide anion levels [25,26,27,28], and increased hyperactivation as well as premature acrosomal reaction [29]. However, most of these studies were performed in whole semen samples [25,27] or in cryopreserved spermatozoa [26,28] and thus investigated a possible effect of the toxicant when present in semen or in the male genital tract. Spermatozoa may be exposed to PAHs also in the female genital tract. Indeed, BaP metabolites and their adducts have been found in cervical mucus and follicular fluids, being higher in female smokers [30,31], as well as in ovarian tissue [32]. After deposition of semen in the vagina, spermatozoa undergo an immediate selection process through cervical mucus, a process that can be mimicked in vitro with the swim-up procedure. 

The aim of this study was to evaluate the in vitro effects of different concentrations of BaP (1, 5, 10 µM) on both unselected and swim-up selected spermatozoa after 3 and 24 h of incubation. In particular, we evaluated the effects of BaP on the main sperm functions (motility, hyperactivation, ability to penetrate a viscous medium, and acrosome reaction) and its effects on oxidative stress, DNA fragmentation, and apoptosis.

## 2. Results

### 2.1. BaP Effect on Sperm Motility and Viability

**Figure 1:**
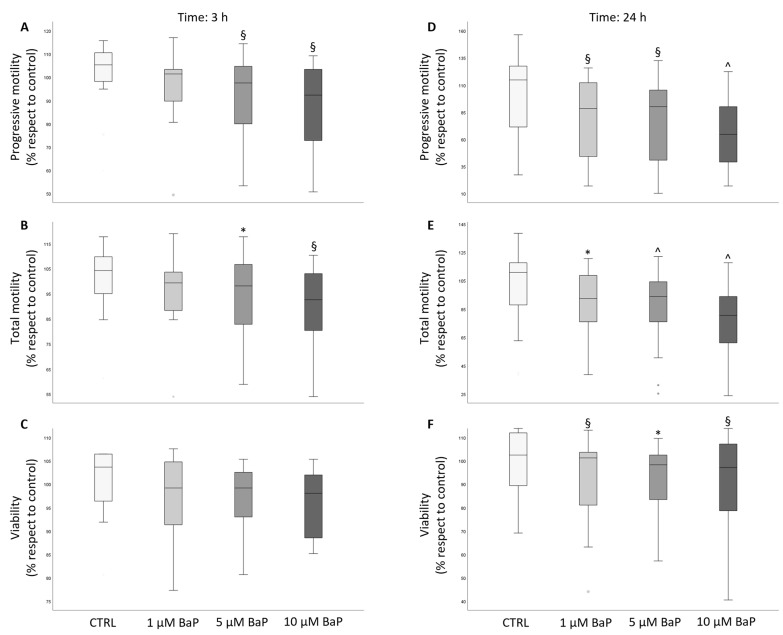
Box plots representing the median values of progressive and total motility and viability in the control and BaP-treated (1, 5, and 10 µM) swim-up selected spermatozoa after 3 ((**A**–**C**) panels, respectively, *n* = 12) and 24 ((**D**–**F**) panels, respectively, *n* = 18) h of incubation. Values are reported as a percentage with respect to the control. * *p* < 0.05, § *p* < 0.01, ^ *p* < 0.001 vs. CTRL.

**Figure 2:**
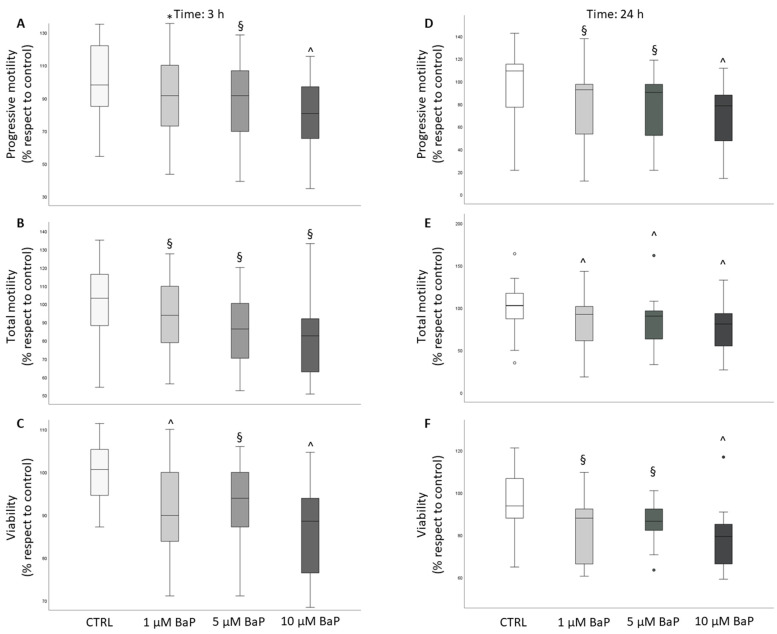
Box plots representing the median values of progressive and total motility and viability in the control and BaP-treated (1, 5, and 10 µM) unselected spermatozoa after 3 ((**A**–**C**) panels, respectively, *n* = 12) and 24 ((**D**–**F**) panels, respectively, *n* = 20) h of incubation. Values are reported as a percentage with respect to the control. * *p* < 0.05, § *p* < 0.01, ^ *p* < 0.001 vs. CTRL.

**Figure 3:**
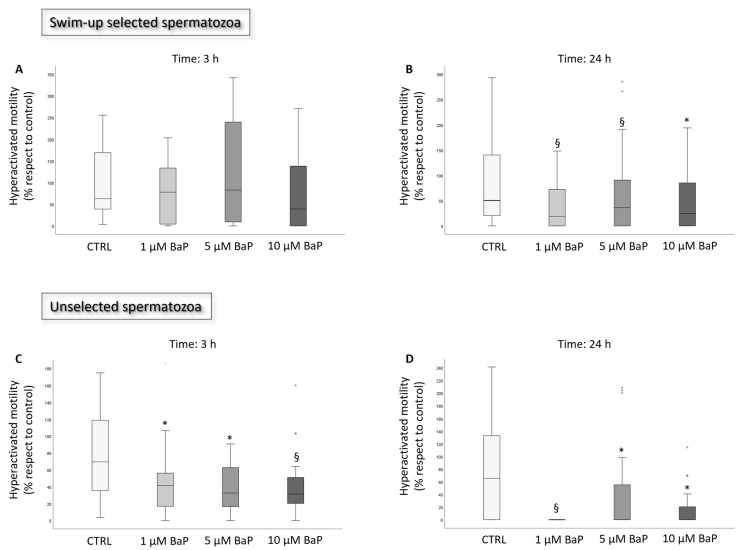
Box plots representing the median values of hyperactivated motility in the control and BaP-treated (1, 5, and 10 µM) swim-up selected and unselected spermatozoa after 3 (panel (**A**) (*n* = 8) and (**C**) (*n* = 19), respectively) and 24 (panel (**B**) (*n* = 24) and (**D**) (*n* = 23), respectively) h of incubation. Values are reported as a percentage with respect to the control. * *p* < 0.05, § *p* < 0.01, vs. CTRL.

After incubation with BaP, progressive and total motility were significantly reduced in swim-up selected and unselected spermatozoa both after 3 (Figure 1A,B and Figure 2A,B) and 24 h (Figure 1D,E and Figure 2D,E) of incubation. In unselected spermatozoa, BaP significantly reduced sperm viability at both incubation times and with all tested concentrations (Figure 2C,F). A slight, not significant decrease in sperm viability was observed after 3 h of treatment of swim-up selected spermatozoa (Figure 1C), which became significant with prolonged incubation to 24 h with all the three doses (Figure 1F). A significant decrease in the percentage of hyperactivated motility was observed after 3 and 24 h of incubation with all the three concentrations of BaP in unselected spermatozoa (Figure 3B), whereas a declining effect on hyperactivation was observed in swim-up selected spermatozoa only after 24 h of incubation (Figure 3A,B).

### 2.2. BaP effect on Sperm Ability to Penetrate a Viscous Medium

**Figure 4:**
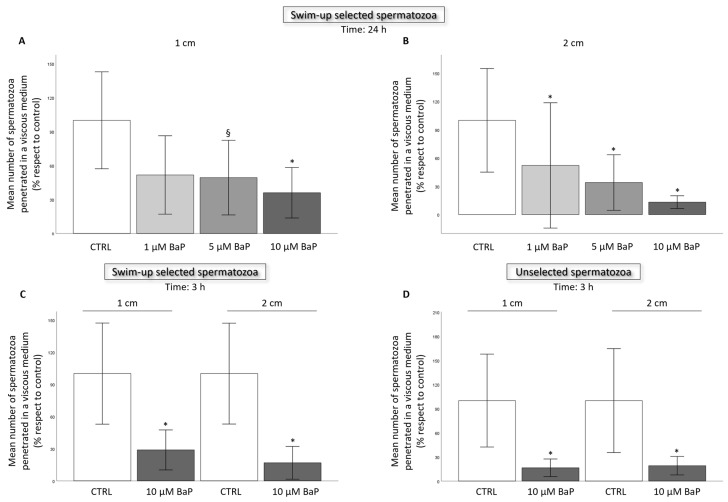
Histograms representing mean ± sd number of spermatozoa penetrated in a viscous medium at 1 and 2 cm (panels (**A**,**B**), *n* = 4) in the control and BaP-treated (1, 5 and 10 µM) swim-up selected spermatozoa after 24 h of incubation. Panels (**C**,**D**) (*n* = 5) show swim-up and unselected spermatozoa after 3 h of incubation with the maximum dose at 1 and 2 cm, respectively. Values are reported as a percentage with respect to the control. * *p* < 0.05, § *p* < 0.01, vs. CTRL.

After 24 h of incubation of swim-up selected with 1, 5, and 10 µM BaP, we observed a significant decrease in the number of spermatozoa penetrated in the viscous medium at 1 and 2 cm (Figure 4A,B). Incubation with 10 µM for 3 h reduced sperm penetration at 1 and 2 cm both in swim-up selected and unselected spermatozoa (Figure 4C,D). 

### 2.3. BaP Effect on Acrosome Reaction

**Figure 5:**
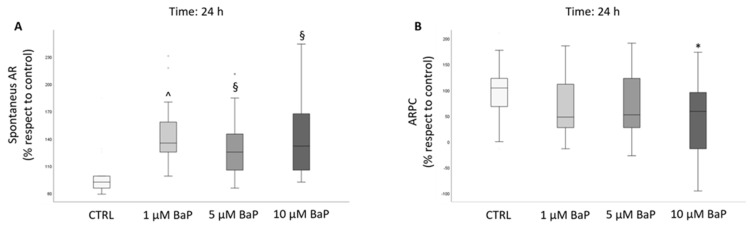
Box plots representing median values of spontaneous (panel (**A**), *n* = 14) and progesterone-induced AR (calculated as ARPC, panel (**B**), *n* = 14) in the control and BaP-treated (1, 5, and 10 µM) swim-up selected spermatozoa after 24 h of incubation. Values are reported as a percentage with respect to the control. * *p* < 0.05, § *p* < 0.01, ^ *p* < 0.001 vs. CTRL.

We next evaluated the spontaneous and progesterone-induced acrosome reaction (AR) after incubation of swim-up selected spermatozoa with the pollutant. A conspicuous increase in the percentage of spontaneous AR was observed after 24 h of incubation with all doses of BaP (Figure 5A). A significant inhibition of the response to progesterone (calculated as the acrosome reaction following progesterone challenge, ARPC) was evident only after incubation for 24 h with 10 µM BaP (Figure 5B).

### 2.4. BaP Effect on DNA Fragmentation, Sperm Oxidative Status, and Apoptosis

**Figure 6:**
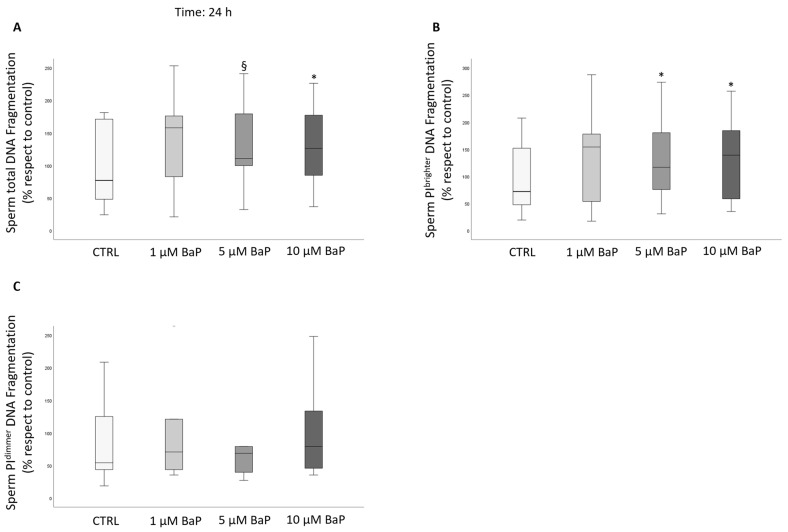
Box plots representing median values of total (**A**) PI brighter (**B**) and PI dimmer (**C**) DNA fragmentation (*n* = 9) in the control and BaP-treated (1, 5, and 10 µM) swim-up selected spermatozoa after 24 h of incubation. Values are reported as a percentage with respect to the control. * *p* < 0.05, § *p* < 0.01, vs. CTRL.

Incubation with 5 and 10 µM BaP for 24 h significantly increased the percentage of DNA fragmentated spermatozoa in total and PI brighter, but not PI dimmer populations (Figure 6).

DNA fragmentation may occur either due to the induction of an apoptotic pathway or an increase in reactive oxidative species (ROS) [33]. 

**Figure 7:**
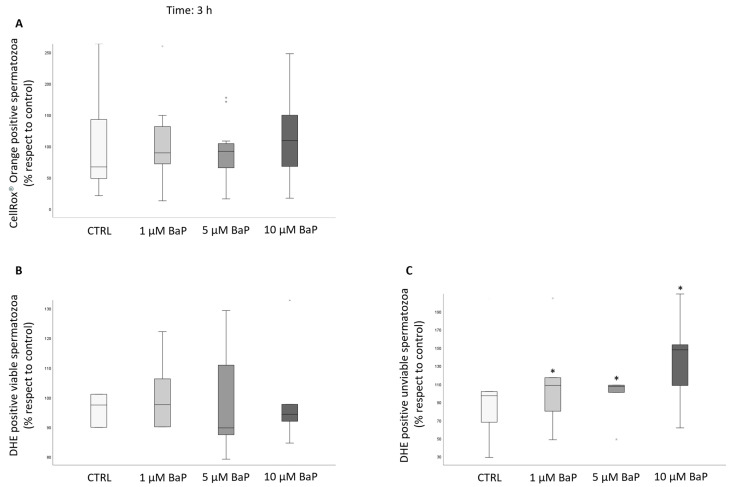
Box plots representing median values of CellROX^®^ Orange (panel (**A**), *n* = 5) and DHE positivity (panels (**B**,**C**), *n* = 5) of viable and unviable swim-up selected spermatozoa in the control and BaP-treated (1, 5, and 10 µM) after 3 h of incubation. Values are reported as a percentage with respect to the control. * *p* < 0.05 vs. CTRL.

To study sperm intracellular ROS, we used two different probes [34] after 3 h of incubation with BaP of swim-up selected spermatozoa. By staining the samples with CellROX^®^ Orange, which detects ROS only in viable spermatozoa, the percentage of positive events did not differ among the control and the BaP treated samples (Figure 7A). The same result was observed in viable spermatozoa by using Dihydroethidium (DHE, Figure 7B). On the contrary, the percentage of unviable spermatozoa which were positive with respect to DHE increased with respect to the control with all doses of BaP (Figure 7C).

**Figure 8:**
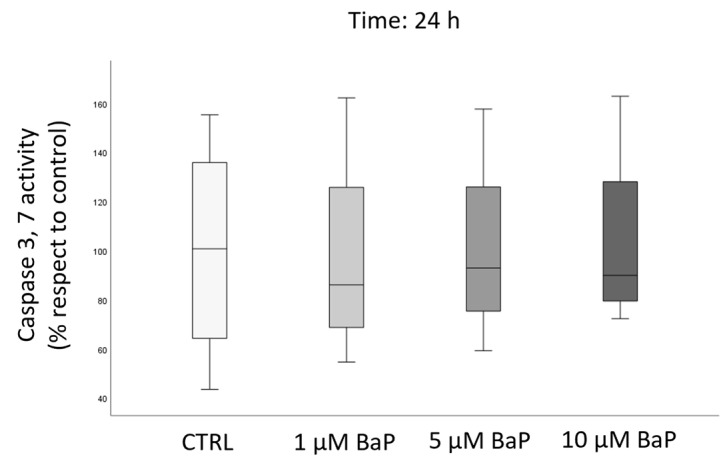
Box plots representing median values of caspase-3 and -7 activity (*n* = 4) in the control and BaP-treated (1, 5, and 10 µM) swim-up selected spermatozoa after 24 h of incubation. Values are reported as a percentage with respect to the control.

Next, it was evaluated if the toxicant is able to induce apoptotic cascade determining caspase-3 and -7 activity. No differences were observed between the control and BaP-treated samples after 24 h (Figure 8). 

## 3. Discussion

This study demonstrates that several human sperm functions are disrupted in vitro by incubation with BaP, a PAH widely diffused in the environment [10]. Furthermore, we show that BaP induces sperm DNA fragmentation (sDF) and increases the percentage of spermatozoa showing intracellular oxidative signs. Interestingly, these effects have also been demonstrated in swim-up selected spermatozoa, i.e., after an in vitro processing mimicking the selection occurring in the female genital tract after deposition in the vagina, there is an indication that the toxicant may also alter functions in spermatozoa showing good motility and morphological characteristics. In particular, the toxicant reduced the percentage of spermatozoa achieving a hyperactivated motility, which is necessary to penetrate the oocyte vestments and, importantly, the ability to penetrate a viscous medium in a capillary both in selected and unselected spermatozoa. Evaluation of the ability to penetrate a viscous medium in a capillary somehow reflects the difficulties encountered by spermatozoa during their journey in the female genital tract [35]; therefore, our results suggest that the presence of the toxicant in semen or in female genital fluids may compromise the process of natural fertilization. 

PAHs undergo a metabolic activation in human body and PAHs and their metabolites have been demonstrated in urine [36,37], seminal fluid [24], cervical mucus [30], ovarian cells [38], and follicular fluid [31,39]. PAH levels in semen and, in particular, those in BaP, differ between fertile and idiopathic infertile males (where BaP has been found, on average, at about 0.2 µM levels) [24]. We show here that BaP reduces both motility and viability of unselected spermatozoa washed from seminal plasma, even after short-term incubations (3 h) and low (1 µM) concentrations of the toxicant, leading to a reduction in the number of spermatozoa able to penetrate a viscous medium. Considering that, according to the study of Nayak et al. [24], the sum of all 13 PAHs evaluated in semen of idiopathic unexposed infertile men may largely exceed 10 µM. Therefore, most of these substances have been shown to affect testicular and sperm functions in vivo and in vitro [40,41,42]. Consequently, the synergistic effects of BaP with other PAHs [28] or other toxicants [43] cannot be excluded, and our study reinforces the hypothesis that these environmental pollutants may be responsible of sperm alterations leading to subfertility. In addition, the demonstration of high levels and toxic effects of semen PAHs suggest that, in case of use for in vitro fertilization, spermatozoa should be washed as soon as possible from seminal fluid to avoid detrimental effects. 

As previously mentioned, PAHs, including BaP, are also found in fluids of the female genital tract [31,39] and have been shown to disrupt embryo development and alter ovary function [32,44]. We show here that the toxicant has detrimental effects in vitro, also in swim-up selected spermatozoa, reducing many functions requested to reach and fertilize the oocyte during natural conception (for review see [45]), such as the ability to migrate in a viscous medium, hyperactivation, and inducing a massive AR. Physiological AR likely occurs during crossing of the cumulus matrix [46,47] and the occurrence of BaP in female genital fluids might compromise such function, inducing a premature AR. An increase in AR in swim-up selected spermatozoa has also been shown by Mukhopadhyay et al. [29], employing much higher concentrations of BaP (up et 100 µM). Furthermore, we show here that BaP induces an increase in the percentage of DNA fragmented spermatozoa. Elevated levels of sDF may have several detrimental effects on reproduction, including alterations in embryo development, implantation, and induction of miscarriage [48]. Previous studies demonstrated a negative effect of BaP on sDF by using different methodological approaches. In particular, Sipinen et al. [26] found an increase in sDF, which was evaluated with the Comet assay on the total sperm population after incubation with 10 and 25 µM BaP levels. Similarly, Alamo et al. [27] found an increase in sDF, which was assessed using Tunel on the total sperm population after incubation with 15 and 45 µM BaP. Here, we also show that 5 and 10 µM BaP induce DNA fragmentation in the brighter PI population of swim-up selected spermatozoa, i.e., in those spermatozoa which may be viable, motile, and with normal morphology [49] as well as a higher probability to participate in the fertilization process. Although such detrimental effects have been obtained in vitro with BaP concentrations that exceed those found in follicular fluids and women sera [31,39], we cannot exclude that they may be present in vivo considering that other PAHs, with possible synergistic effects, may be present in the female genital tract. 

An important aspect concerns the mechanism(s) responsible for BaP detrimental effects on spermatozoa. We investigated the possible role of induction of oxidative stress by evaluating intracellular ROS with two probes, CellROX^®^ Orange, which shows specificity for H_2_O_2_ only in viable spermatozoa and DHE, which detects both H_2_O_2_ and O_2_^−^ species both in viable and unviable spermatozoa [34,50]. We found that all the three tested concentrations of BaP increased the percentage of ROS-positive unviable swim-up selected spermatozoa as detected with DHE after 3 h of incubation, likely reflecting the slight decrease in viability detected in the same sample (Figure 1C). No increase was observed in viable spermatozoa with both probes. In a previous study, we demonstrated that CellROX^®^ Orange- and DHE-positive viable spermatozoa are associated with good semen characteristics and do not show apoptotic features, reflecting a sperm fraction related to better performances [34]. We hypothesized that most ROS-positive viable spermatozoa show physiological ROS levels [34] likely supporting their physiological functions [51,52]. We can infer that BaP, by increasing intracellular ROS to non-physiological levels, may lead to sperm death in a small fraction of swim-up selected spermatozoa. BaP does not appear to activate an apoptotic pathway in swim-up selected spermatozoa as no increase in effective caspase activity was found in our study. Previous studies, performed in vitro in different cell types and different experimental conditions, demonstrated a positive effect of BaP on oxidative stress and apoptosis [14,53,54,55,56]. Regarding in vitro effects on human spermatozoa, Alamo et al. [27] found an increase both in the percentages of mitochondrial O_2_^−^ membrane lipid peroxidation and apoptotic spermatozoa after 3 h of stimulation with BaP, employing different methodological approaches and using higher concentration (15–45 µM) of the toxicant. Clearly, more studies are needed in order to understand whether induction of oxidative stress and/or apoptosis are involved in the detrimental effects of BaP on spermatozoa. 

In conclusion, our in vitro study suggests that BaP present in semen and in the female genital fluids may affect reproductive functions of human spermatozoa. In particular, we show for the first time that the toxicant may interfere with sperm swimming up in viscous fluids similar to those encountered within the female genital tract. 

## 4. Materials and Methods

### 4.1. Chemicals

Human tubal fluid (HTF) medium and human serum albumin (HSA) were purchased from Fujifilm Italia S.p.A. (Milan, Italy). An In Situ Cell Death Detection Kit was purchased from Roche Molecular Biochemicals (Milan, Italy). Vybrant™ FAM Caspase-3 and -7 Assay, and CellROX^®^ Orange Reagent and Dihydroethidium were purchased from Invitrogen by Thermo Fisher Scientific (Waltham, MA, USA). Methylcellulose 4000 cP (1% *w*/*v*) was purchased from Sigma–Aldrich and capillary tubes in borosilicate glass (0.20 mm × 2.0 mm × 50 mm) were from VitroCom (Mountain Lakes, NJ, USA). Benzo[a]pyrene was purchased from Merck Life Sciences S.r.l. (Milan, Italy). BaP was prepared by dissolving as a stock solution in dimethyl sulphoxide (DMSO) at 50 mM. Yo-Pro-1 (Y1) and Propidium Iodide (PI) were purchased from Life Technologies. 

### 4.2. Human Semen Samples

Semen samples (*n* = 47) were consecutively obtained by masturbation after a minimum of two and a maximum of seven days of sexual abstinence, according to World Health Organization laboratory manual for the examination and processing of human semen [57]. Thirty minutes after semen collection, the volume, viscosity, and pH of the sample were evaluated. Sperm concentration, motility, and morphology were determined according to WHO 2010 [57]. Sperm concentration was evaluated by an improved Neubauer chamber after dilution in formalin containing buffer. The percentages of progressive, non-progressive, and immotile spermatozoa were evaluated on 200 spermatozoa using an optical microscope (Nikon Eclipse Ci) with a 40× objective and a 37 °C heated plate. Sperm viability was evaluated using an eosin test. 

The experiments were performed on washed semen samples (after centrifugation at 500× *g* for 10 min and reconstitution of the pellet in HTF −10% HSA at the concentration of 10 × 10^6^/mL) and after swim-up selection. To perform swim-up, 1 mL of HTF −10% HSA was gently layered on an equal volume of semen sample and incubated at 37 °C. After 50 min, 800 μL of the upper medium phase containing the motile fraction of spermatozoa was collected. Only those samples with a progressive motility ≥ 90% after selection were used for the experiments. 

To evaluate the BaP effects on human spermatozoa, each semen sample was divided into four aliquots of 4 × 10^6^ spermatozoa. An aliquot was incubated with HTF-10% HSA containing the solvent (DMSO) at the maximum concentration (0.5%) and three remaining aliquots with 1, 5, and 10 µM concentration of BaP for 3 or 24 h at 37 °C, 5% CO_2_. 

BaP doses were chosen based on concentrations used in in vitro studies [18,19,26,28].

After incubation, progressive and total motility, viability, kinetic parameters and hyperactivation, sDF, AR, caspase-3 and -7 activity, oxidative stress, and penetration of artificial viscous medium were analyzed.

### 4.3. Assessment of Sperm Motility and Viability

Sperm motility was assessed using a phase-contrast microscope equipped with a 40× objective, which could observe at least 200 spermatozoa. Motility was scored as the percentage of progressive, non-progressive, and immotile spermatozoa. Sperm viability was evaluated by using eosin–nigrosin staining on at least 200 spermatozoa discriminating between pink-colored cells (dead) and uncolored sperm (viable). 

### 4.4. Assessment of Caspase-3 and -7 Activity

Caspase activity was evaluated using Vybrant^TM^ FAM Caspase-3 and -7 Assay Kit based on a fluorescent inhibitor of caspase (FLICA™), according to Marchiani et al. [58]. After incubation with the toxicant, each sample (4 × 10^6^ spermatozoa) was washed, resuspended in 600 µL of PBS and split into two aliquots. In the test sample, 10 μL of 30× FLICA working solution was added, while the negative control was incubated only with PBS medium. After 1 h of incubation at 37 °C, samples were washed with Wash Buffer 1× and fixed with 40 μL of 10% formaldehyde for 10 min at room temperature. Wash and fixative solutions were supplied by the kit. Sperm samples were washed again twice and resuspended in 400 µL of Wash Buffer 1× containing 6 µL of propidium iodide solution (PI, 50 µg/mL in PBS). Samples were acquired with flow cytometry. 

### 4.5. Assessment of Sperm DNA Fragmentation (sDF)

sDF was detected using the TUNEL/PI method, as previously described [49], by using In Situ Cell Death Detection Kit fluorescein (Roche Molecular Biochemicals, Milan, Italy). 

After incubation for 24 h with BaP, fixed spermatozoa (10 × 10^6^ spermatozoa) were centrifuged at 500× *g* for 5 min and washed twice with 200 µL of PBS with 1% BSA. Then, spermatozoa were permeabilized with 0.1% Triton X-100 in 100 µL of 0.1% sodium citrate for 4 min in ice and the samples were divided into two aliquots for a labelling reaction. After one washing, a test sample was incubated in 50 µL of labeling solution (supplied by the kit) containing the TdT enzyme (1:10) for 1 h at 37 °C in the dark, whereas the negative control was incubated only with the labelling solution. Later, samples were washed twice, resuspended in 500 µL of PBS-BSA, stained with 10 µL of Propidium Iodide (PI, 30 mg/mL), and incubated in the dark for 10 min at room temperature. Samples were analyzed with flow cytometry. 

### 4.6. Assessment of Oxidative Stress

The effect of BaP on intracellular ROS species was evaluated in swim-up selected spermatozoa. Intracellular ROS were detected both by CellROX^®^ Orange Reagent and DHE as described previously [34]. After incubation with BaP, spermatozoa were resuspended in 400 µL HTF −10% HSA and divided into two equal aliquots. One aliquot was incubated with CellROX^®^ Orange (1 µM) for 30 min at 37 °C and 5% CO_2_ or DHE (1.25 µM) for 20 min at room temperature. For the negative control, the other aliquot was incubated with only medium in the same experimental conditions. After incubation, three washes with PBS were carried out and the samples were resuspended in 300 µL of PBS. Yo-Pro-1 (Y1, 25 nM) was added for acquisition with flow cytometry. 

### 4.7. Flow Cytometry Acquisition and Analysis

Samples were acquired with a FACScan flow cytometer (BD Biosciences, San Jose, CA, USA) and were equipped with a 15-mW argon-ion laser for excitation by including 8000 events in the characteristic forward scatter/side scatter region of spermatozoa [49]. Green fluorescence of FLICA, TUNEL and Y1 and red fluorescence of CellROX^®^ Orange, DHE, and PI were revealed with the FL-1 (515–555 nm wavelength band) and FL-2 (563–607 nm wavelength band) detector, respectively. In the dot plot of fluorescence distribution of the negative controls a marker, including 99% of total events, was established and then translated in the corresponding test sample. All the events beyond the marker were considered positive. Analysis of data was performed with the CellQuest-Pro software program version 5.2.1 (BD Biosciences, Franklin Lakes, NJ USA). 

### 4.8. Assessment of Kinetic Parameters and Hyperactivation

After incubations, samples were analyzed by using a C.A.S.A. (Computer-Assisted-Sperm Analysis) system (Hamilton Thorn Research, Beverly, MA, USA) to evaluate kinetic parameters and hyperactivation. The settings used during C.A.S.A. procedures were as follows: analysis duration of 1 s (30 frames); maximum and minimum head size, 50 and 5 µm^2^; minimum head brightness, 170; minimum tail brightness, 70 [34]. 

To calculate the fraction representing the percentage of hyperactivated spermatozoa (HA, %) we manually set the following threshold values: VCL ≥ 150 µm/s, ALH ≥ 7 µm and LIN ≤ 50% [59]. A minimum of 200 motile cells and 5 fields were analyzed for each aliquot.

### 4.9. Assessment of Acrosome Reaction (AR)

After swim-up selection, spermatozoa were incubated for 2 h at 37 °C to induce capacitation. Then, the aliquots were incubated with BaP and acrosome reaction was determined by staining with FITC-labeled Arachis hypogea (peanut) lectin using fluorescent microscopy [60]. Both the control and treated samples were divided into two aliquots. An aliquot was incubated with progesterone (10 µM) and the other one with 0.1% DMSO (vehicle control) for 1 h at 37 °C. Afterward, spermatozoa were washed using centrifugation and resuspended in 500 µL of hypo-osmotic swelling medium (HOS) to evaluate AR only in live spermatozoa. After 1 h at 37 °C, spermatozoa were washed again and fixed in 100 µL of ice-cold methanol. The sperm were layered on a slide, air-dried, and stored at −20 °C. For acrosome staining, sperm were incubated for 20 min in the dark, with lectin and green fluorescence being observed under an Axiolab A1 FL fluorescence microscope (Carl Zeiss, Jena, Germany) equipped with filter set 49 and an oil immersion 100× magnification objective. For each condition, 200 cells with curled tail (viable) were analyzed for their acrosomal status. 

### 4.10. Assessment of Sperm Penetration in Artificial Viscous Medium

Penetration into methylcellulose (4000 cP, 1%, *w*/*v*) was assessed as described previously [35]. Artificial viscous medium was obtained by dissolving 10 mg of methylcellulose in 1.5 mL of HTF and mixing it overnight at room temperature. Methylcellulose was introduced in glass capillary tubes (0.20 mm × 2.0 mm × 50 mm) with force capillarity after incubation for 15 min. One end of the capillarity tube was sealed with plasticine and the other end was cut before to be placed in 200 µL of a washed semen sample or swim-up selected spermatozoa after incubation with different doses of BaP. The sample was inclined to 45° and motile spermatozoa were allowed to migrate into the penetration medium for 2 h at 37 °C and 5% CO_2_. Lastly, a capillary tube was wiped to remove residual spermatozoa from the surface of the glass and then analyzed with a microscope. The number of spermatozoa migrated at 1 and 2 cm was counted in 3 fields and the average number/field was calculated. 

### 4.11. Statistical Analysis 

Statistical analysis was performed using the Statistical Package for the Social Sciences version 28.0 (SPSS, Chicago, IL, USA) for Windows. The Kolmogorov–Smirnov test was used to test the data distribution. Data are expressed as mean (±s.d.) when normally distributed and as median (interquartile, IQR) when non-normally distributed. All values are reported as a percentage with respect to the control. To compare groups, Student’s *t*-test, for paired data and normally distributed parameters, or the Wilcoxon signed-rank test, for non-normally distributed parameters, were used. A *p*-value of 0.05 was considered significant. 

### 4.12. Ethical Approval

This study was approved by local ethical committee (Ref: CEAVC Em. 2019-353—Study 1286) after obtaining informed consent to use the remaining semen after completion of the analysis. Semen samples of patients undergoing routine semen analysis at the Andrology Laboratory of Careggi University Hospital of Florence were used. Only semen samples from patients (*n* = 47) with parameters above the fifth percentile according to the 5th edition of WHO [57] and without detectable leukocytes were included. 

## Data Availability

Data are unavailable due to privacy and ethical restrictions.
